# Sleep for Children

**Published:** 1870-09

**Authors:** 


### SLEEP FOR CHILDREN.

All know how unwelcome it is to have to get up in the morning when waked out of a sound sleep; an effort when we wake spontaneously, and yet parents are sometimes extremely impatient, become very angry and resort to harsh words, and harsher blows, if a child does not instantly rise from bed when waked up. The old who can't sleep after daylight, have no patience with their children whom they find in bed several hours later, forgetting that growing children under twenty ought to be in bed nine or ten hours out of the twenty-four, and by so doing, will add to their chances for a long and healthful life.

A striken mother writes, "Friends tell me if you allow your son of seventeen to lie in bed so late, he will be always weakly." But they give no proof of the fact. In Genesis, God "rested from all his works;" in the New Testament, the Saviour of mankind, "being wearied with his journey, sat down by the well." Some seem to think that it is a waste of time, a sin, to be doing nothing; that if there is no employment for the hands, a book should be taken up; thus making of glorious life one incessant work and toil and drag. We get the power to work by rest and sleep; there are sins of omission as well as of commission; not to do right is as blameworthy as to do wrong. Doing too much and doing nothing are equal suicides; recreation is as much a necessity as application, and they are wisest who enjoy as well as work.

